# Muscle strength and foot pressure vary depending on the type of foot pain

**DOI:** 10.1038/s41598-024-56490-8

**Published:** 2024-03-11

**Authors:** Jin Hyuck Lee, Jangsun Hwang, Hyungyu Park, Hyunoh Kang, Wonkyu Song, Doo-Ah Choi, Cheul-Hun Seong, Woo Young Jang

**Affiliations:** 1 Department of Sports Medical Center, Anam Hospital, Korea University College of Medicine, Seoul, Republic of Korea; 2 Department of Orthopedic Surgery, College of Medicine, Korea University, 73, Inchon-ro, Seongbuk-gu, Seoul, 02841 Republic of Korea; 3 Institute of Nanobiomarker-Based Medicine, Korea University, Seoul, Korea; 4 LG Electronics Inc, Seoul, Korea; 5 Huraypositive Inc, Seoul, Korea

**Keywords:** Metatarsalgia, Plantar fasciitis, Muscle strength, Foot pressure, Foot posture, Health care, Signs and symptoms

## Abstract

This study compared muscle strength and foot pressure among patients with metatarsalgia, patients with plantar fasciitis, and healthy controls. A total of 31 patients with foot pain (14 metatarsalgia and 17 plantar fasciitis) and 29 healthy controls participated in the study. The strengths of the plantar flexor and hip muscles were measured using isokinetic and handheld dynamometers, respectively. Foot pressure parameters, including the pressure–time integral (PTI) and foot arch index (AI), were assessed using pedobarography. Compared with the healthy control group, plantar flexor strength was significantly reduced in the affected feet of the metatarsalgia and plantar fasciitis groups (F = 0.083, all *p* < 0.001); however, hip strength was significantly decreased only in the affected feet of the metatarsalgia group (F = 20.900, *p* < 0.001). Plantar flexor (*p* < 0.001) and hip (*p* = 0.004) strength were significantly lower in the metatarsalgia group than in the plantar fasciitis group. The PTI was lower in the forefeet of the affected feet in the metatarsalgia (*p* < 0.001) and plantar fasciitis (*p* = 0.004) groups. Foot AI (*p* < 0.001) was significantly reduced only in the metatarsalgia group. These results suggest the need to consider the evaluation of muscle strength and foot pressure in both feet for the diagnosis and treatment of foot pain.

## Introduction

Foot pain is highly prevalent at various ages and is especially common in middle-aged and older women^1,2^. Foot pain can lead to a variety of musculoskeletal problems and is associated with decreased activities of daily living, impaired balance, and an increased risk of falls^1–3^. Hence, the diagnosis and management of foot pain are important for increasing physical activity and improving quality of life.

In middle-aged and older women, the most common cause of foot pain is plantar fasciitis^4^, followed by metatarsalgia^5^. Plantar fasciitis is commonly observed in flat feet and often causes pain in the heel owing to excessive stretching of the plantar fascia^6,7^. In contrast, metatarsalgia is commonly observed in the cavus foot, and pain often occurs in the forefoot in the region of the metatarsal heads^7^ owing to restricted joint mobility^8^. Foot pain is often determined based on pain location and foot posture. Indeed, foot posture affects foot kinematics during walking^8,9^, which may affect lower-extremity muscle function^10–14^ and foot pressure^15^. A recent study^12^ reported differences in lower-extremity muscle strength and foot pressure between patients with plantar fasciitis and flatfoot and those with a normal foot. Another study^9^ reported differences in foot pressure among normal, flat, and cavus feet and the association of foot posture with foot pressure during gait. However, the reasons for the differences in muscle strength, foot pressure, and foot posture among patients with metatarsalgia and those with plantar fasciitis, especially compared with healthy controls, are not clear. To the best of our knowledge, no studies have directly compared muscle strength, foot pressure, and foot posture among patients with metatarsalgia, patients with plantar fasciitis, and healthy controls. Moreover, study data on metatarsalgia are limited.

Therefore, the present study compared muscle strength, foot pressure, and foot posture among patients with metatarsalgia, patients with plantar fasciitis, and healthy controls. We hypothesized that patients with metatarsalgia and plantar fasciitis would show decreased muscle strength, increased foot pressure, and different foot postures compared with healthy controls.

## Methods

### Study design and setting

This prospective comparative case–control study followed the Strengthening the Reporting of Observational Studies in Epidemiology (STROBE) guidelines for non-pharmacological treatments and obtained informed consent from all patients. This study was conducted from July 2018 to August 2022 and was approved by our Institutional Review Board (2018AN0168). Outpatients presenting for physiotherapy with a primary report of foot pain were screened according to the eligibility criteria. Among a total of 109 enrolled patients, only those with forefoot or rearfoot pain were selected by an orthopedic surgeon. Metatarsalgia and plantar fasciitis were evaluated by physical examination, plain radiography, and ultrasonography. The inclusion criteria were pathological effusion in the metatarsophalangeal joints for metatarsalgia^16^ and fascia thickening of > 4 mm for plantar fasciitis^17^. A total of 78 patients with affected muscle strength, foot pressure, and walking were excluded for the following reasons: (1) bilateral foot pain; (2) other concomitant foot pain (i.e., hallux valgus and Achilles tendonitis); (3) knee osteoarthritis with Kellgren–Lawrence grade > 2; 4) previous foot and ankle surgeries; (5) inability to perform the tests owing to pain; (6) neurogenic deformities; (7) administration of analgesics and anti-inflammatory drugs within 4 weeks or injections in the past 6 months; (8) gastrocnemius and hamstring muscle tightness; and (9) anatomical problems of the feet such as plantar plate tears, plantarflexed metatarsal bone, and metatarsal length discrepancy. Finally, 31 patients with foot pain (14 metatarsalgia and 17 plantar fasciitis) participated in this study. We also included 29 healthy controls with no history of ankle or foot trauma, or foot pain symptoms were selected from our database and agreed to participate in this study (Table 1).

**Table 1:** Demographic data of the study participants by group.

	Metatarsalgia (n = 14)	Plantar fasciitis (n = 17)	Healthy control (n = 29)	*p*-value
Sex (male/female)	5/9	6/11	10/19	
Age (years)^a^	57 ± 11.2	54 ± 9.4	57 ± 6.9	0.421
Height (cm)^a^	165 ± 4.3	166 ± 7.1	161 ± 7.4	0.618
Weight (kg)^a^	69.1 ± 12.6	71.1 ± 8.2	67.4 ± 10.4	0.364
Body mass index (kg/m^2^)^a^	25.0 ± 3.4	25.8 ± 2.5	26.0 ± 3.8	0.112
Injured side (right/left)	8/6	11/6		
Pain VAS (point)	5.1 ± 0.8	4.9 ± 1.1		

*VAS* visual analog acale.

^a^Values are expressed as mean ± standard deviation.

### Measurements and outcome variables

#### Muscle strength

At our institution, the strengths of the plantar flexor and hip muscles were measured in patients with foot pain using an isokinetic dynamometer and a handheld dynamometer, respectively. Plantar flexor strength was evaluated for five repetitions of plantar flexion at 30°/s using an isokinetic device (Biodex Multi-Joint System 4, Biodex Medical Systems, Inc.) for each leg with each participant in a semi-seated position and 20° knee flexion^12^.

Isometric hip muscle strength was measured using a handheld dynamometer (microFET2, Hoggan Scientific, LLC, Salt Lake City, UT, USA, 2016) with 45° flexion, 20° abduction of the hip joint, and 90° flexion of the knee joint with the participants lying on their side. The evaluation was performed twice and the average value was used^12^.

Muscle strength was calculated in this study by normalizing the peak torque to body weight. Plantar flexor and hip muscles strength were recorded as Nm kg^−1^ × 100 and kgf/kg, respectively. The intraclass correlation coefficients (ICCs) for plantar flexor and hip muscle strength were 0.84 and 0.87, respectively.

#### Foot pressure and foot posture

Foot pressure was measured using pedobarography (Tekscan, MA, USA) and recorded at 50 Hz while walking for 2 m. All participants were evaluated after stepping on the pressure platform for three steps on the affected foot while walking the 2 m distance. The foot pressure parameters were evaluated using the pressure–time integral (PTI).

PTI was defined as the time integral of the mean pressure (N/cm^2^ s) in each of the three areas (forefoot, midfoot, and rearfoot) during walking^18^, and is a better indicator than peak pressure^19^.

Foot posture was assessed using the foot arch index (AI)^9,20^. The foot AI was calculated as the entire footprint area divided by the area of the middle third of the footprint (AI = B/A + B + C), and has demonstrated acceptable reliability and validity.^20^ Based on a previous study^20^, the cut-off scores for foot AI were defined as follows: high-arched (< 0.21), normal (0.21–0.28), and low-arched (> 0.28). The high- and low-foot AI scores were defined as low-arched and high-arched foot postures, respectively, in the present study.

### Statistical analysis

The sample size calculation was based on a > 10% difference in plantar flexor muscle strength between groups, a significance α level of 0.05, and a power (1–β) of 0.8^12^, which was considered clinically significant. To determine the sample size, we conducted an a priori power analysis using one-way analysis of variance (ANOVA), which revealed that a minimum sample size of 36 participants was sufficient to detect a 10% difference in plantar flexor strength between the groups (effect size f(V): 0.567). The power required to detect the differences in muscle strength was 0.837.

We used the Shapiro–Wilk test to verify normal data distributions. The Kruskal–Wallis test was performed to compare demographic data between the groups. One-way ANOVA was performed to determine significant differences in all outcome variables between the groups. When between-group comparisons were made, Tukey’s correction was applied as a post hoc test (*p* < 0.017). Correlations among pain visual analog scale (VAS), muscle strength, foot pressure, and foot AI were assessed using Pearson’s correlation coefficients. Data were analyzed using IBM SPSS Statistics for Windows, version 20.0 (IBM Corp., Armonk, NY, USA). The significance level was set at α = 0.05.

### Ethics approval and consent to participate

Korea University Anam Hospital approved this study (2018AN0168). The study was performed in accordance with the ethical standards as laid out in the 1964 Declaration of Helsinki. Informed consent was obtained from all individual participants included in the study.

## Results

Participant age, weight, height, or body mass index (*p* > 0.05) did not differ significantly between groups.

### Comparisons of muscle strength between the groups

**Figure 1:**
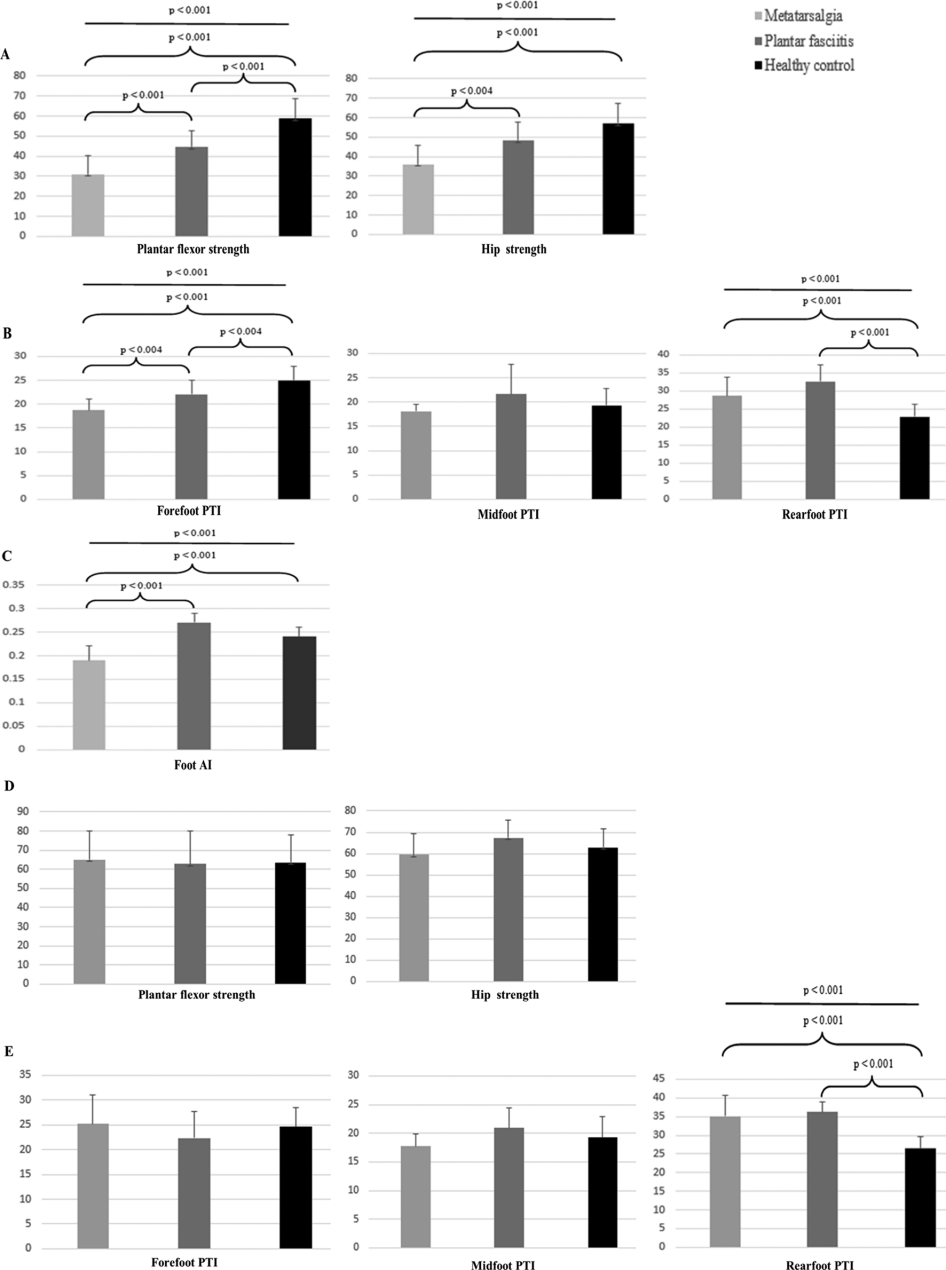
Comparisons of muscle strength, foot pressure, and foot AI among the three groups. (**A**–**C**) affected feet; (**D**, **E**) unaffected feet. PTI, pressure–time integral; AI, arch index.

In the affected feet (Table 2 and Fig. 1), plantar flexor (F = 44.175, *p* < 0.001) and hip (F = 20.900, *p* < 0.001) strengths differed significantly between the groups. Following the post hoc test, compared with the healthy control group, the plantar flexor strength was significantly lower in the metatarsalgia (58.8 ± 9.9 vs. 31.1 ± 9.0; 95% confidence interval [CI], 20.3 to 35.1; *p* < 0.001) and plantar fasciitis (58.8 ± 9.9 vs. 44.7 ± 8.0; 95% CI 7.1–21.0; *p* < 0.001) groups, whereas hip strength was significantly decreased only in the metatarsalgia (57.1 ± 10.4 vs. 36.1 ± 9.8; 95% CI 12.9 to 29.1; *p* < 0.001) group. Plantar flexor (44.7 ± 8.0 vs. 31.1 ± 9.0; 95% CI 5.4–21.9; *p* < 0.001) and hip (48.4 ± 9.5 vs. 36.1 ± 9.8; 95% CI 3.4–21.3; *p* = 0.004) strength were significantly lower in the metatarsalgia group compared with that in the plantar fasciitis group.

**Table 2:** Comparisons of muscle strength outcomes among the three groups.

Variables	Metatarsalgia	Plantar fasciitis	Healthy control	F	P^1^	P^2^
	M ± SD	M ± SD	M ± SD			
Plantar flexor strength (unaffected)	65.0 ± 15.2 (56.3 to 73.8)	62.8 ± 17.2 (53.9 to 71.6)	63.6 ± 14.6 (58.0 to 69.1)	0.083	0.921	I = 1.0 II = 1.0 III = 1.0
Plantar flexor strength (affected)	31.1 ± 9.0 (25.8 to 36.3)	44.7 ± 8.0 (40.6 to 48.8)	58.8 ± 9.9 (54.9 to 62.5)	44.174	** < 0.001**	**I < 0.001** **II < 0.001** **III < 0.001**
Hip strength (unaffected)	59.4 ± 9.8 (53.7 to 65.0)	57.4 ± 8.1 (53.1 to 61.5)	62.9 ± 8.9 (59.5 to 66.3)	2.229	0.117	I = 1.0 II = 0.137 III = 0.700
Hip strength (affected)	36.1 ± 9.8 (30.3 to 41.7)	48.4 ± 9.5 (43.5 to 53.3)	57.1 ± 10.4 (53.1 to 61.0)	20.900	** < 0.001**	**I = 0.004** II = 0.019 **III < 0.001**

*M* mean, *SD* standard deviation, *P*^*1*^ one-way analysis of variance (ANOVA), *P*^*2*^ post hoc analysis.

I = metatarsalgia group vs. plantar fasciitis group.

II = plantar fasciitis group vs. healthy control group.

III = metatarsalgia group vs. healthy control group.

The measurement units of muscle strength for the plantar flexor and hip muscles were Nm kg^*−*1^ × 100 and kgf/kg, respectively.

Significant values are in bold.

### Comparisons of foot pressure and AI between groups

In affected feet (Table 3 and Fig. 1), the PTI differed significantly in the forefoot (F = 24.211, *p* < 0.001) and rearfoot (F = 31.105, *p* < 0.001) between groups. Following the post hoc test, compared with the healthy control group, PTI was low in the forefoot in the metatarsalgia (25.0 ± 2.9 vs. 18.7 ± 2.4; 95% CI 4.0–8.5; *p* < 0.001) and plantar fasciitis (25.0 ± 2.9 vs. 22.1 ± 2.9; 95% CI 0.6–5.2; *p* = 0.004) groups, but significantly higher in the rearfoot (metatarsalgia: 22.9 ± 3.4 vs. 28.7 ± 5.1; 95% CI −9.2 to −2.5; plantar fasciitis: 22.9 ± 3.4 vs. 32.7 ± 4.6; 95% CI −13.0 to −6.7; all *p* < 0.001). In particular, PTI in the forefoot (18.7 ± 2.4 vs. 22.1 ± 2.9; 95% CI 0.2–7.7; *p* = 0.004) was significantly lower in the metatarsalgia group compared with that in the plantar fasciitis group.

**Table 3:** Comparison of PTI outcomes among the three groups.

Variables	Metatarsalgia	Plantar fasciitis	Healthy control	F	P^1^	P^2^
	M ± SD	M ± SD	M ± SD			
Forefoot-PTI (affected)	18.7 ± 2.4 (17.4 to 20.1)	22.1 ± 2.9 (20.6 to 23.7)	25.0 ± 2.9 (23.9 to 26.1)	24.211	** < 0.001**	**I = 0.004** **II = 0.004** **III < 0.001**
Forefoot-PTI (unaffected)	25.2 ± 5.8 (21.9 to 28.6)	22.3 ± 5.5 (19.5 to 25.1)	24.6 ± 3.9 (23.1 to 26.0)	1.680	0.196	I = 0.223 II = 0.288 III = 0.903
Midfoot-PTI (affected)	18.1 ± 1.4 (17.2 to 18.9)	21.6 ± 6.2 (18.4 to 24.8)	19.3 ± 3.5 (17.9 to 20.6)	2.924	0.062	I = 0.059 II = 0.187 III = 0.628
Midfoot-PTI (unaffected)	17.7 ± 2.2 (16.5 to 19.0)	20.9 ± 3.5 (19.2 to 22.7)	19.2 ± 3.7 (17.8 to 20.6)	3.590	0.034	I = 0.027 II = 0.204 III = 0.390
Rearfoot-PTI (affected)	28.7 ± 5.1 (25.8 to 31.7)	32.7 ± 4.6 (30.3 to 35.1)	22.9 ± 3.4 (21.6 to 24.2)	31.105	** < 0.001**	I = 0.030 **II < 0.001** **III < 0.001**
Rearfoot-PTI (unaffected)	35.1 ± 5.5 (31.9 to 38.3)	36.2 ± 2.8 (34.8 to 37.7)	26.4 ± 3.2 (25.2 to 27.6)	47.317	** < 0.001**	I = 0.696 **II < 0.001** **III < 0.001**

*PTI* pressure–time integral, *M* mean, *SD* standard deviation, *P*^*1*^ one-way ANOVA, *P*^*2*^ post hoc.

I = metatarsalgia group vs. plantar fasciitis group.

II = plantar fasciitis group vs. healthy control group.

III = metatarsalgia group vs. healthy control group.

The measurement unit of the PTI was N/cm^2^ s.

Significant values are in bold.

In the unaffected feet, PTI of the rearfoot differed significantly among the groups (F = 47.317, *p* < 0.001). Following the post hoc test, compared with that of the healthy controls, PTI in the rearfoot was significantly higher in the metatarsalgia (26.4 ± 3.2 vs. 35.1 ± 5.5; 95% CI −11.8 to −5.8; *p* < 0.001) and plantar fasciitis (26.4 ± 3.2 vs. 36.2 ± 2.8; 95% CI −12.7 to −7.0; *p* < 0.001) groups. The PTI did not differ significantly between the metatarsalgia and plantar fasciitis groups.

Foot AI (F = 28.019, *p* < 0.001) was significantly reduced in the metatarsalgia group compared with that in the plantar fasciitis (0.19 vs. 0.27; 95% CI −0.1 to 0; *p* < 0.001) and healthy control (0.19 vs. 0.24; 95% CI −0.1 to 0; *p* < 0.001) groups, but did not differ significantly between the plantar fasciitis and healthy control groups (Fig. 1, *p* > 0.017).

### Correlations between pain VAS, muscle strength, foot pressure, and foot AI

Pain VAS in the metatarsalgia and plantar fasciitis groups showed a significant negative correlation with plantar flexor strength in the affected feet (r = −0.423, *p* = 0.018) and a positive correlation with PTI in the rearfoot of the unaffected feet (r = 0.529, *p* = 0.002, Table 4).

**Table 4:** Pain VAS bivariate correlation by Pearson correlation.

Parameters		VAS	
Plantar flexor strength (affected)	PCC (r)		−0.423
	p-value		**0.018**
Rearfoot-PTI (unaffected)	PCC (r)		0.529
	p-value		**0.002**

Significant values are in bold.

*PCC* Pearson’s correlation coefficient, *VAS* Visual Analog Scale, *PTI* pressure–time integral.

## Discussion

This study compared muscle strength, foot pressure, and foot posture among patients with metatarsalgia, patients with plantar fasciitis, and healthy controls. Our findings showed the weakest plantar flexor and hip muscle strength in patients with metatarsalgia. Metatarsalgia was also associated with low forefoot pressure. Furthermore, patients with metatarsalgia and plantar fasciitis had high pressures in the rearfoot of both feet. Finally, foot posture differed only in the metatarsalgia group.

The plantar fasciitis group in this study showed significantly reduced plantar flexor strength compared with that in the healthy control group. This result is consistent with those previously reported^21,22^. However, the reasons why the plantar flexor and hip muscle strength decreased more in the metatarsalgia group than in the plantar fasciitis and control groups are not known. One possible explanation may involve compensatory strategies to reduce forefoot pain. The forefoot should be used to increase plantar flexor strength. However, patients with metatarsalgia may compensate for increased pain when using their forefeet during the muscle strength test. Similarly, the plantar flexor^23–25^ and hip^24,26^ muscles are mainly used for single-leg stance and forward propulsion during walking. However, patients with metatarsalgia may use compensatory strategies to reduce the activity of the plantar flexors and hip muscles to reduce pain during these movements. Therefore, our findings also showed decreased forefoot-PTI in patients with metatarsalgia compared with the plantar fasciitis and healthy control groups, indicating a lack of sufficient foot adaptation for forward propulsion during walking. However, further studies are needed to clarify whether plantar flexor and hip muscle weaknesses are the cause or result of metatarsalgia.

In this study, rearfoot-PTI was significantly higher in the metatarsalgia and plantar fasciitis groups than in the healthy control group. One possible explanation for this finding is foot posture. Previous studies^19,27^ have reported increased rearfoot-PTI in patients with foot pain and an association between foot posture and foot pain and plantar pressure. Our results showed that metatarsalgia was associated with cavus foot posture (foot AI < 0.21, high-arched foot) and differed from heathy controls. Burns et al.^19^ and Buldt et al.^9^ reported higher rearfoot-PTI in the cavus foot than in healthy controls owing to a lack of load bearing in the midfoot, and that this foot posture was related to foot pain^14^. Another possible explanation may be weakness of the plantar flexor muscles. Such weakness may lead to a lack of ankle moment, which may alter walking patterns. Ong et al.^25^ and Bruel et al.^28^ suggested that plantar flexor muscle weakness may cause heel walking, indicating a higher load on the heel. Furthermore, in this study, compared with the healthy controls, the PTI was higher in the rearfoot of feet unaffected by metatarsalgia and plantar fasciitis. Although the reason for this finding is unclear, it may be explained by the overuse of the contralateral foot owing to an altered walking pattern owing to foot pain. Sullivan et al.^29^ reported that patients with foot pain change their walking patterns as a strategy to reduce pain. Both feet are used synchronously while walking; therefore, biomechanical stress on one side can naturally increase the use of the other. Therefore, patients with unilateral foot pain may have bilateral foot problems^30^. Arie et al.^31^ reported that biomechanical alterations accounted for > 90% of all etiological factors. Taken together, the results of this study and those of previous studies may explain to the higher plantar pressure on the rearfoot in patients with metatarsalgia and plantar fasciitis.

### Clinical implication

The results of this study revealed decreased plantar flexor and hip muscle strength in the following order: patients with metatarsalgia < patients with plantar fasciitis < healthy controls. Metatarsalgia may be a compensatory strategy for reducing pressure on the forefoot. Additionally, the metatarsalgia and plantar fasciitis groups showed increased rearfoot pressure. Moreover, the metatarsalgia group showed a high-arched foot posture, whereas the plantar fasciitis group showed a posture similar to that in the healthy control group. Hence, caution is needed when diagnosing these diseases based solely on foot posture. Previous studies reported no difference in foot posture between individuals with and without foot pain^32,33^. Therefore, whether the results in this study are the cause or result of metatarsalgia or plantar fasciitis is unknown; however, our results suggest that muscle strength and foot pressure should be evaluated in both feet for the diagnosis and treatment of foot pain.

### Limitations

This study has some limitations. First, the intrinsic foot muscles were not measured. These muscles play important roles in foot posture and function. Previous studies^10,34–36^ reported that alterations in the function of the intrinsic foot muscles owing to changes in foot posture may lead to variation in foot posture and function. Therefore, further studies on intrinsic foot muscles are needed to clarify the results of the present study. Second, although walking speed can affect foot pressure results^37^, we did not evaluate this factor. Third, we did not assess neural subsystems. McKeon et al.^10^ reported that foot core systems such as the passive (ligament and plantar fascia), active (foot muscles), and neural (neuromuscular control) subsystems, play significant roles in normal foot function. Therefore, assessment of neuromuscular control abilities such as dynamic postural stability^34^ should be considered in patients with foot pain. Finally, randomized controlled trials with larger sample sizes are required to validate our results.

## Conclusion

Compared with the healthy controls, patients with foot pain, such as metatarsalgia and plantar fasciitis, showed decreased plantar flexor and hip muscle strength. Foot pressure was low in the forefoot of the affected foot, but high in the rearfoot of the affected and unaffected feet. In particular, compared with patients with plantar fasciitis and the healthy controls, patients with metatarsalgia demonstrated weaker plantar flexor and hip muscles, low forefoot pressure, and high foot posture.

## Data Availability

The data that support the findings of this study are available from author, Jin Hyuck Lee but restrictions apply to the availability of these data, which were used under license for the current study, and so are not publicly available. Furthermore, all data generated or analyzed during the current study will not be disclosed due to policy of the Korea University Anam Hospital Research Ethics Board.
